# Performance of ACR-TIRADS in assessing thyroid nodules does not vary according to patient age

**DOI:** 10.1007/s42000-024-00585-4

**Published:** 2024-07-19

**Authors:** Andrea Leoncini, Marco Curti, Lorenzo Ruinelli, Elena Gamarra, Pierpaolo Trimboli

**Affiliations:** 1 Servizio Di Radiologia E Radiologia Interventistica, Istituto Di Imaging Della Svizzera Italiana (IIMSI), Ente Ospedaliero Cantonale (EOC), 6900 Lugano, Switzerland; 2 Servizio Di Endocrinologia E Diabetologia, Ospedale Regionale Di Lugano, Ente Ospedaliero Cantonale (EOC), 6900 Lugano, Switzerland; 3 Team Data Science & Research, Ente Ospedaliero Cantonale, Area ICT, 6500 Bellinzona, Switzerland; 4 Clinical Trial Unit, Ente Ospedaliero Cantonale (EOC), Bellinzona, Switzerland; 5 Facoltà Di Scienze Biomediche, Università Della Svizzera Italiana (USI), 6900 Lugano, Switzerland

**Keywords:** Thyroid, TIRADS, FNAC, Age, Young, Elderly

## Abstract

**Aims:**

A few studies have evaluated the performance of the American College of Radiology Thyroid Imaging Reporting And Data System (ACR-TIRADS) in pediatric and elderly patients and found differences between the latter two age groups and middle adulthood. Thus, the present study was undertaken to explore the possible variation of ACR-TIRADS performance across different ages of patients.

**Methods:**

A retrospective population undergoing thyroidectomy was selected to use histology as the reference standard. Ultrasound images were reviewed, and alignment of ACR-TIRADS with the corresponding histological diagnosis was made afterwards. Results of the age groups were compared. The ACR-TIRADS diagnostic performance was calculated considering the assessment of nodules across risk categories (i.e., from TR1 to TR5), rate of unnecessary FNAC (UN-FNAC), and rate of necessary but non-performed FNAC (NNP-FNAC).

**Results:**

Overall, 114 patients with a total of 220 nodules (46 carcinomas) were included. The rate of UN-FNAC was 66.3%, being 93.1% in TR3, 82.1% in TR4, and 31.4% in TR5. There were 15 NNP-FNACs. No significant difference was observed between age groups in terms of sample size, nodule, cancer, and FNAC. The nodule assessment according to ACR-TIRADS categories did not vary across ages. Sensitivity and specificity recorded in three age tertiles were not significantly different.

**Conclusions:**

The present study shows that the performance of ACR-TIRADS is not significantly influenced by patient age.

## Introduction

Thyroid nodules (TNs) occur at all ages and are often detected incidentally [1]. Since the vast majority of TNs are benign, and bearing in mind that most TN patients are asymptomatic, a uniform and precise diagnostic work-up is usually applied worldwide with the aim of ruling out TNs at negligible risk of cancer from further diagnostic and therapeutic procedures [2]. From this point of view, ultrasonography (US) holds a pivotal role. US is well recognized as the most accurate first-line imaging procedure for discriminating between benign and malignant TNs. Over the last few years, international societies have published modified US-based risk stratification systems, generally referred to as Thyroid Imaging Reporting and Data System (TIRADS), to improve the reliability of US [3]. TIRADS was basically conceived to standardize terminology, assess TNs across risk categories, and indicate if there is need for fine-needle aspiration cytology (FNAC). It has been demonstrated that TIRADSs are accurate, with performance comparable to that of FNAC [4], and they may also be depended upon to point to “unnecessary” FNAs, biopsies in other words that may not be indicated [5].

Age is always a determinant of the risk for a large number of diseases [6], with, for example, old age (late adulthood, the elderly) being a risk factor for cancer and non-communicable diseases, while young people are at risk for other pathological conditions. In general, aging is associated with factors influencing the risk for developing comorbidities within a complex process wherein diseases and comorbidities may influence the onset of each other [7]. Moreover, the same disease may have different clinical presentation, progression, and aggressiveness according to patient age. Based on these premises, it has been hypothesized that TIRADS accuracy may vary according to patient age. In the case of thyroid cancer, and in particular papillary carcinoma (PTC), which represents the most common malignant endocrine tumor, TIRADSs have proven their high accuracy in detecting this type of thyroid cancer at all ages [8]. However, as age is known to be an independent prognostic factor in PTC patients (i.e., age > 55 year means a higher risk of relapse [9]), PTC US presentation can well change according to age. This concept led the authors to reconsider the performance of TIRADSs in age-specific settings. On one hand, evidence-based data indicate that TIRADS accuracy in the pediatric population is lower than what was initially expected [10–12]. On the other hand, although TIRADSs seem to be quite reliable in the elderly, their performance varies, with lower accuracy in this age group in some studies [13, 14]. These findings suggest that, as in other contexts in the field of the thyroid, age should be included in TIRADSs to better tailor patient management. Efforts have been made over the last few years to further improve the performance of TIRADS using artificial intelligence (AI) studies and other imaging applications [15–17]. More specifically, currently, a major project involving international societies is ongoing with the aim of creating a universal TIRADS (I-TIRADS) [18].

**Fig. 1:**
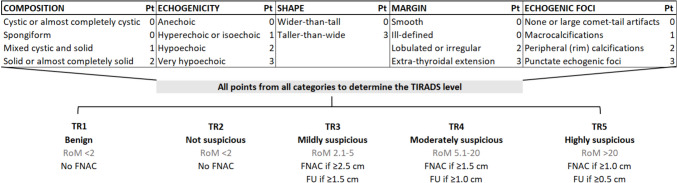
Ultrasound characteristics and risk categories of ACR-TIRADS with associated risk of malignancy (RoM) and recommended actions. Legend. Nodules are assigned to a specific category after evaluation of their composition, echogenicity, shape, margin, and echogenic foci. Points are given for all the US features. The point total determines the nodule’s category

The present study was undertaken to explore the possible variation of TIRADS performance across different patient ages. The American College of Radiology TIRADS (ACR-TIRADS), as the most popular among the TIRADSs, was selected for study (Fig. 1) [19]. A retrospective population undergoing thyroidectomy was included.

## Material and methods

### Setting

Our institution is the cantonal public healthcare organization and includes four regional hospitals while also representing the center with the highest number of thyroid surgeries. Moreover, most thyroid histologies are read by our pathology institute and, therefore, the institutional database includes records of almost all cases of thyroid surgery performed in our region.

### Case selection

The study includes cases from January 2019 to June 2023. Patients with a diagnosis of malignancy were classified as PTC, while follicular thyroid carcinoma (FTC) and medullary thyroid carcinoma (MTC) were staged according to the current TNM edition [20]. The study protocol was composed of five steps, as follows. (1) A retrospective search was undertaken for adults belonging to the categories “adulthood” and “middle adulthood” undergoing thyroidectomy for all causes in the institutional database: this phase was performed by a data scientist blinded to the clinical data. (2) Patients with available preoperative thyroid US images in RIS-PACS were included: this phase was performed by two radiologists. (3) Inclusion of all TN > 5 mm and re-classification of them according to ACR-TIRADS. Nodules with diameter < 5 mm were excluded to avoid difficult US interpretation in lesions with negligible clinical impact [2, 19]: this phase was performed by the same two radiologists, separately and blinded to the final diagnosis. (4) TIRADS data and corresponding histological diagnosis were aligned by an expert endocrinologist. (5) Demographic data of patients were extracted by the same data scientist blinded to the clinical data. This five-step strategy was chosen with the aim of using histology instead of FNAC as the reference standard and to overcome as much as possible selection and performance bias.

### Age subgroups

Patients included in the study were divided into several age groups in order to explore as accurately as possible the age effect. A dichotomization was first made into two groups, i.e., > or < 55 years, which represents an unfavorable prognostic factor for PTC recurrence [9, 20, 21], and > or < 65 years, which is defined as the seniority threshold [22]. In addition, the entire series was divided into tertiles and the three subgroups were compared with each other.

### Measures

TIRADS diagnostic performance was calculated considering the rate of unnecessary FNAC (UN-FNAC) indicated according to ACR-TIRADS (Fig. 1) [19]. Since ACR-TIRADS recommends performing FNAC in TNs classified as TR3, TR4, or TR5 and having a dimension greater than the category-specific size threshold (i.e., > 2.5 cm, > 1.5 cm, and > 1.0 cm, respectively), these cases were scored as FNAC+ , while the remaining cases were scored as FNAC-. Accordingly, the rate of UN-FNAC was calculated as FNAC+ with benign histology/FNAC+. Cancers deemed FNAC- were scored as FNAC necessary but non-performed (NNP-FNAC).

### Statistical analysis

Continuous parameters were expressed as median and interquartile range (IQR) and compared using the Mann–Whitney test. Their Gaussian distribution was analyzed using the D’Agostino-Pearson normality test. Frequencies of subgroups were compared using the chi-square test. Receiver operating characteristic (ROC) curve analysis was performed, the area under the curve (AUC) was calculated to estimate the performance of ACR-TIRADS in different age groups, and the best cut-off point (employing the Youden Index) was identified. Accordingly, sensitivity, specificity, positive likelihood ratio (+LR), and negative likelihood ratio (-LR) were calculated. ROC curves were compared according to DeLong’s test. The statistical significance level was set at *p* < 0.05. Statistical analyses and figures were performed with GraphPad Prims version 7 (GraphPad software, CA, USA) or MedCalc (MedCalc Software Ltd, Belgium).

### Ethics

This study was approved by the local Ethics Committee.

## Results

### Characteristics of the study population

A total of 114 patients were included, 84 females and 30 males, with median age 55 (44–66) years. In total, US images of 220 TNs (median major diameter 18 mm, IQR 11–29) were reviewed, with 46 thyroid carcinomas found among them. Specifically, there were 36 PTCs, nine FTCs, and one MTC.

### TN assessment according to ACR-TIRADS

According to ACR-TIRADS categories, seven (3.2%) were TR1, 46 (20.1%) TR2, 76 (34.5%) TR3, 50 (22.7%) TR4, and 41 (18.6%) TR5. Cancer prevalence was 1/53 TR1-TR2, 6/76 TR3, 10/50 TR4, and 29/41 TR5. The rate of FNAC+ was 29/76 in TR3, 28/50 in TR4, and 35/41 in TR5. The rate of UN-FNAC was 27/29 (93.1%) in TR3, 23/28 (82.1%) in TR4, and 11/35 (31.4%) in TR5. Overall, UN-FNACs were 61/92 (66.3%). Finally, there were 15 NNP-FNACs, one classified as TR1, four as TR3, five as TR4, and five as TR5.

### Performance of ACR-TIRADS across the patient lifespan

**Fig. 2:**
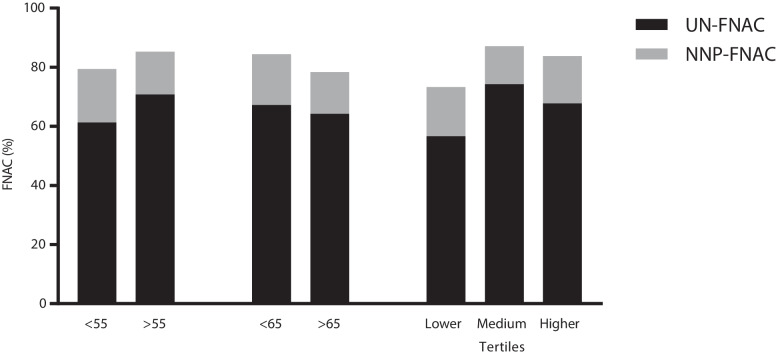
Frequency of UN-FNAC and NNP-FNAC in age groups. Legend – The percentage is calculated according to all cases with an indication for FNAC (FNAC+)

**Fig. 3:**
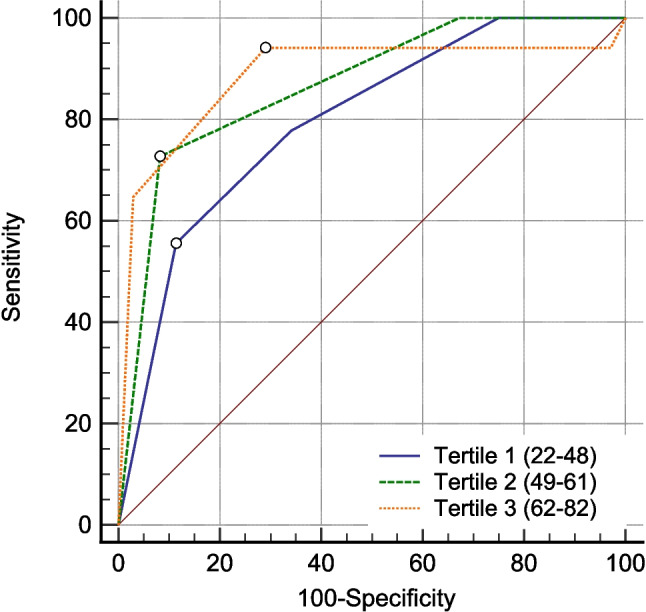
ROC curve analysis considering the three age tertiles. Legend – The three tertiles are defined in the text. Data of sensitivity, specificity, and AUC of the three age groups are presented in Table 2

Both patient-based and lesion-based analysis were performed. The performance of ACR-TIRADS was evaluated considering the distribution of TNs and cancers, and according to the rate of UN-FNAC and NNP-FNAC (Table 1). Figure 2 illustrates the percentage of UN-FNAC and NNP-FNAC in age groups. According to the study design, age groups were comparable to each other. Overall, no significant difference was found between groups in terms of sample size, nodule, cancer, and FNAC data, with some exceptions. The TN size of patients in the top tertile was significantly smaller than that of patients in the second tertile (*p* = 0.048). The frequency of cancer types was significantly different between patients < or > 65 years of age (*p* = 0.047). The main result was that the assessment of TNs and cancers according to ACR-TIRADS categories did not vary across ages. Figure 3 depicts the ROC curves of the tertiles. The comparison between three ROC curves did not show any significant difference (T1 vs. T2 *p* = 0.35, T1 vs. T3 *p* = 0.28, and T2 vs. T3 *p* = 0.82), when the ACR-TIRADS criteria was category 3 in the older tertile and 4 in the remaining two tertiles (Table 2). Regarding FNAC indication, the number of FNACs recommended among patients > 65 years was significantly higher than that of older patients (*p* = 0.042).

**Table 1:** Characteristics of age-adjusted subgroups and comparison with each other

	Age 55 years		Age 65 years		Tertiles of age		
	< 55	≥ 55	< 65	≥ 65	T1 (22–48)	T2 (49–61)	T3 (62–82)
Patients							
N	55	59	84	30	38	38	38
Female/male	43/12	41/18	62/22	20/8	30/8	27/11	27/11
Nodules							
N	96	124	153	67	62	73	85
Size, median (IQR)	20 (13–31)	17 (11–27)	19 (13–31)	16 (10–25.2)	20.5 (11.7–31.7)	19 (13.5–31)	16.5 (10–25)*
TR5	24	17	29	12	15	14	12
Cancers							
N	25	21	32	14	18	12	16
Size, median (IQR)	15 (12–28.5)	16 (11.5–34.5)	15 (11.5–29.7)	16.5 (11.7–32.5)	18.5 (12.5–32.2)	15 (10.7–32.2)	15.5 (11.2–27)
PTC/FTC/MTC	22/3/0	14/6/1	28/4/0	8/5/1*	17/1/0	9/3/0	10/5/1
TR1	-	1	-	1	-	-	1
TR2	-	-	-	-	-	-	-
TR3	5	1	6	-	4	2	-
TR4	5	5	7	3	4	1	5
TR5	15	14	19	10	10	9	10
FNAC							
FNAC+	44	48	64	28*	30	31	31
UN-FNAC	27	34	43	18	17	23	21
NNP-FNAC	8	7	11	4	5	4	6

Legend. IQR, interquartile range. FNAC+ , indication for FNAC. FNAC-, no indication for FNAC. UN-FNAC, unnecessary FNAC. NNP-FNAC, necessary but not performed FNAC. PTC, papillary thyroid carcinoma. FTC, follicular thyroid carcinoma. MTC, medullary thyroid carcinoma. Size is expressed as mm. *Significant difference

**Table 2:** Data of predictivity tests recorded in the three age tertiles

	ACR-TIRADS criterion	Sensitivity (95%CI)	Specificity (95%CI)	+ LR	-LR	AUC
T1	> 4	55.56 (30.8–78.5)	88.64 (75.4–96.2	4.89	0.50	0.79
T2	> 4	75.00 (42.8–94.5)	90.00 (78.2–96.7)	7.50	0.28	0.86
T3	> 3	93.75 (69.8–99.8)	71.01 (58.8–81.3)	3.23	0.088	0.88

Legend. The three age tertiles are defined in the text. CI, confidence interval. LR, likelihood ratio. AUC, area under the curve

## Discussion

Both the prevalence and the course of human diseases are influenced by patient age. In the field of thyroid cancer, age is a well-recognized prognostic risk factor, patients with differentiated thyroid carcinoma (DTC) older than 55 years having a higher risk of dying from DTC [20, 21] and developing structural recurrence [9] than younger age groups. In addition, DTC among the pediatric population has a different biology from that of adults [23]. This is so relevant that age was proposed as the pivotal feature during the initial assessment of the risk of recurrence of DTC [24]. Thus, according to the above concepts, it is reasonable to consider that the performance of TIRADSs may change with age. The present study reports interesting new findings that warrant extensive discussion.

First, despite some slight differences between age groups, the performance of ACR-TIRADS in discriminating benign from malignant TNs did not significantly change across the patient lifespan. In fact, both the distribution by category of TNs and the rate of FNAC indication were not significantly different between young and elderly patients. Moreover, the rate of UN-FNAC and NNP-FNAC also remained unchanged over the patient lifespan. Second, the frequency of cancers observed in the age groups was quite similar. In addition, there were no difference between groups in gender, TN size, and cancer size. On the other hand, a mildly significant difference was found in cancer types between patients older than 65 years versus adult patients of younger age (mostly middle adulthood).

Besides a few studies including either young or elderly patients [10–14], literature on the age effect on TIRADS is scant. A recent study by Walter et al. which evaluated whether cancer prevalence changes according to Bethesda FNAC categories found that the rate of malignant FNAC decreases as the age of the patient increases. [25]. Moreover, they observed that age significantly influences the malignancy rates of ACR-TIRADS. An interesting example of this is that the cancer rate of TR5 of patients aged 20–39 years, 40–59 years, and ≥ 60 years was 64.7%, 45.9%, and 22.6%, respectively. Even if the latter study used FNAC as the reference standard with possible selection bias, the results are of particular interest at this time when the I-TIRADS project is ongoing [18]. The present study is not likely to confirm those data since it is probable that different case selection used (i.e., histological reference from a series of patients undergoing surgery) determines apparent discordance between results. However, when we look at FNAC series, we find populations undergoing FNAC for many reasons (i.e., strict indication according to TIRADS and other). In some cases, FNAC may be performed only as a cautionary measure, even if worrisome US signs are lacking. Young people may be incidentally diagnosed with TN, and physicians are prone to require further investigation, especially when the lesion is suspicious. In a retrospective review of US images of patients undergoing surgery, it was noted that these effects usually disappear. In addition, while FNAC series substantially include only PTC that can be recognized on cytology [26], histological series like the present one also include FTC and MTC that are not or are only partially detectable on FNAC [27, 28]. The most accurate ROC-derived threshold of ACR-TIRADS to distinguish benign from malignant nodule observed between the third tertile and the other tertiles (Fig. 3) merits consideration. These data appear to be in line with the different cancer type distribution according to age (Table 1). In fact, the FTC rate in elderly individuals was higher than in younger patients. Since FTC generally presents as isoechoic without typical features of PTC [27], the best cut-off decreased from > 4 in the first two tertiles to > 3 in the third. This is yet more firm proof that the TIRADSs were basically conceived on the basis of PTC US presentation [26]. Most studies based on FNAC series do not include FTC that is usually cytologically indeterminate, has low/intermediate US risk, and then may be infrequently operated on. The latter data can be reported only in histological series like the present one [26]. From this point of view, a slightly different performance of ACR-TIRADS across ages is present. Accordingly, we should consider that elderly people may harbor different types of cancer compared to young patients, resulting in lower TIRADSs reliability. In any case, the cytological and histological series are useful to further extend our knowledge about TIRADS performance, while the upcoming I-TIRADS will benefit from the additional data. To summarize, the present study shows that when we consider all histological series and cancer types, the performance of ACR-TIRADS does not change according to patient age. The present findings represent a novelty in the TIRADS literature.

Ultrasonography is a safe and low-cost procedure and is widely used in TN patients. However, how to improve the accuracy of thyroid US is currently a hot topic. Researchers have indeed studied the limitations of US and indicated that implementing the use of AI may provide greater insight into thyroid disorders thereby improving their treatment [15–17, 29]. At this time, most publications / articles in this field are focused on assessing TN US images related to datasets, despeckling algorithms, segmentation algorithms, and classification algorithms. These data are encouraging and open up new perspectives for clinicians. Furthermore, as investigated in the present study, US and TIRADSs still have non-imaging-related limitations to be resolved. On the whole, TIRADSs are radiological recommendations that do not consider the patient profile (e.g., age, gender, comorbidities, and medications). Our data showed that patient age should not modify the performance of TIRADS, although our findings diverge from those reported by other authors [10–14]. Both our own and previous studies may suffer from selection bias (see above). Furthermore, whether we can currently consider TIRADSs reliable independently of patient age remains to be clarified. In addition, no studies have demonstrated whether TIRADSs can diagnose cancer at an early stage, even considering that ACR-TIRADS indicates FNAC when the TN size is large [30]. From this standpoint, further algorithm-based proposals including patient profile are of particular interest for clinicians. Our advice is that further studies be conducted comparing the performance of humans using TIRADSs versus other imaging-based applications and models. Examples include filtering algorithms [31] or semantic segmentation models based on encoder-decoder architectures or computer-aided diagnosis [32].

The limitations of the present study should be mentioned. First, this is a retrospective analysis of US images of patients undergoing thyroid surgery for any indication. Second, reviewing US images stored in PACS by multiple raters may represent bias, even if discordant assessment was resolved via mutual consultation to achieve a final consensus. Third, pediatric patients were not found in the institutional database during the study period, making analysis of this age group non-feasible. A major strength is that the present study includes only histological diagnosis, thus avoiding the bias of cytological evaluation as a reference standard. Overall, the sample size and the selection strategy should be taken into account as a potential bias of the results. As previously mentioned, we aimed to enroll a series of patients with histological diagnosis to avoid selection bias included in the FNAC series. Even if assuming only histology as a reference standard cannot exclude biases (i.e., a histological series enrolls only patients with indication for surgery), we chose to enroll histological cases to include cancers other than PTC [23–25], increase as much as possible the number of TNs of each patient, and avoid the limitation associated with FNAC-based series. However, the cancer rate that we found (i.e., 46 among 114 patients) may represent an overestimation of the generally anticipated cancer frequency in TN series. Nevertheless, all thyroid carcinomas were included in our series with satisfactory relative frequencies (36 PTCs, nine FTCs, and one MTC) and the TIRADS performance could be explored not only in PTC, as usually occurs in FNAC series.

In conclusion, the present study shows that the performance of ACR-TIRADS is not significantly influenced by patient age across his/her lifespan. More studies are needed to verify these data in order to contribute to paving the way for I-TIRADS.

## Data Availability

The data sets used and/or analyzed during the current study are available from the corresponding author on reasonable request.
